# 

**Published:** 1793

**Authors:** 


### CATALOGUE OF BOOKS.

1. ADVICE to the Female Sex in general, particularly those in a State of Pregnancy and Lying-in: The Complaints incident to their respective Situations are specified, and Treatment recommended, agreeable to modern Practice; the Result of Observation and Experience: To which is added an Appendix, containing some Directions relative to the Management of Children in the first Part of Life. By *John Grigg*, Practitioner in Midwifery, Surgeon to the Pauper Charity in Bath, and late of his Majesty's Navy. 8vo. Bath, 1789.

2. Medical Advice to the Inhabitants of Warm Climates, in the Domestic Treatment of all all the Difeafes incidental therein: With a few ufeful Hints to new Settlers, for the Preservation of Health, and the Prevention of Sicknefs. By *Robert Thomas*, late of the Ifland of Nevis, Surgeon. To the Work are prefixed fome Obfervations on the proper Management of new Negroes, and the general Condition of Slaves in the Sugar Colonies. Alfo are annexed a Lift of the Medicines recommended in the Treatment of the Difeafes, and an explanatory Table of the Weights and Meafures ufed by Apothecaries. 8vo. *Johnfon*, London, 1790.

3. An Effay on the Changes produced in the Body by Operations of the Mind. By the late Dr. *Corp*, of Bath. 8vo. *Ridgway*, London, 1791.

4. Obfervations on Scrophulous Affections, with Remarks on Schirrus, Cancer, and Rachitis. By *Robert Hamilton*, M. D. Fellow of the Royal College of Phyficians, Edinburgh; F. R. S. Edin. Honorary Member of the R. Ph. S. Edin. and C. M. S. London. 12mo. *Dilly*. London, 1791.

5. A remarkable Cafe of Madnefs, with the Diet and Medicines ufed in the Cure. By *William Perfect*, M. D. of Weft Malling, in Kent,

Kent, and Member of the London Medical Society. 8vo. *Evans*, London, 1791.

6. A New Medical Dictionary, or general Repository of Physic. By *G. Motherby*, M.D. C.M.S. The Third Edition, revised and corrected, with considerable Additions, by *G. Wallis*, M.D. S.M.S. Lecturer on the Theory and Practice of Physic in London. Folio. *Johnson*, London, 1791.

7. A New Collection of Medical Prescriptions, distributed into twelve Classes, and accompanied with pharmaceutical and practical Remarks, exhibiting a View of the present State of the Materia Medica and Practice of Physic, both at Home and Abroad. By a Member of the London College of Physicians. 12mo. *Baldwin*, London, 1791.

8. On Electricity; with occasional Observations on Magnetism; pointing out the Inconsistency and Fallacy of the Doctrine of positive and negative Electricity; and investigating and explaining the true Principles, Composition, and Properties, of Electric Atmospheres. By *E. Peart*, M.D. 8vo. *Miller*, London, 1791.

9. A concise History of the Human Muscles, carefully compared with the Subject; collated with the Historia Musculorum of Albinus, and and with the Works of ſeveral other more modern Anatomiſts. Interſperſed with occaſional Inſtructions, particularly calculated to facilitate the Labours of the Diſſector. By *Thomas Wright*, Licentiate of the Royal College of Surgeons, and Superintendant of the Diſſecting Pupils to the ſame. 12mo. Dublin, 1791.

10. The Annals of Chemiſtry, or a Collection of Memoirs relative to Chemiſtry, and the Arts with which it is connected. By Meſſrs. de *Morveau*, *Lavoiſier*, *Monge*, *Berthollet*, *de Fourcroy*, Baron *de Dietrich*, *Haſſenfratz*, and *Adet*. Tranſlated from the French. Vol. I. 8vo. *Johnſon*, London, 1791.

11. Phyſical and Chemical Eſſays: Tranſlated from the original Latin of Sir *Torbern Bergman*, Knight of the Order of Waſa, Profeſſor of Chemiſtry at Upſal, &c. To which are added, Notes and Illuſtrations by the Tranſlator. Vol. III. 8vo. Edinburgh, 1791.

12. A Caſe of Extra-Uterine Geſtation of the Ventral Kind: including the Symptoms of the Patient from the earlieſt Period of Pregnancy to the Time of Death (fifteen Months); with the Appearance upon Diſſection. By *William Turnbull*, A.M. F.M.S. Surgeon. 4to. *Johnſon*, London, 1791, with four Plates.

13. For-

13. Formulæ Medicamentorum concinnatæ; or Elegant Medical Prefcriptions for various Diforders. Tranflated from the Latin of the late Dr. *Hugh Smith*. To which is prefixed a Sketch of his Life. 8vo. *Barr*, London, 1791.

14. A complete Treatife on the Origin, Theory, and Cure of the Lues Venerea, and Obftructions in the Urethra, illuftrated by a great Variety of Cafes. Being a Courfe of twenty-three Lectures, read in Dean Street, Soho, in the Years 1790 and 1791. By *Jeffe Foot*, Surgeon. 4to. *Becket*, London, 1792.

15. An Analyfis of the Mineral Waters of Tunbridge Wells. 8vo. *Murray*, London, 1792.

16. An Effay, Philofophical and Medical, concerning Modern Clothing. By *Walter Vaughan*, M. D. Phyfician at Rochefter, Kent. 8vo. *Robinfons*, London, 1792.

17. A fhort and eafy Introduction to fcientific and philofophic Botany. By *Samuel Saunders*. 12mo. *White*, London, 1792.

18. A fingular Cafe of Reproduction of the Sphincter Ani, and three other Cafes annexed; which illuftrate the Ufe of a frefh Porter Fomentation and Seed Poultice in the Cure of Mortification: Addreffed to *J. Heavifide*, Efq. Sur-

Surgeon Extraordinary to his Majesty, by *Richard Griffith.* 8vo. *Murray*, London, 1792.

19. An Analysis of the London Pharmacopœia, explaining the native Principles, elective Attractions, Qualities, Uses, and Doses, of the various Preparations and Compositions; and particularly calculated for the Use of the junior Students. By *Robert White*, M. D. 8vo. *Cadell*, London, 1792.

20. Medical Histories and Reflections. By *John Ferriar*, M. D. Physician to the Manchester Infirmary and Lunatic Hospital. 8vo. *Cadell*, London, 1792.

21. A Treatise on the Mineral Waters of Harrowgate; containing the History of those Waters, their chemical Analysis, medicinal Properties, and plain Directions for their Use. By *Thomas Garnett*, M. D. Physician at Harrowgate, Member of the Royal Medical, Royal Physical, and Natural History Societies of Edinburgh, and of the Literary and Philosophical Society of Manchester. 8vo. *Johnson*, London, 1792.

22. A Treatise on the Dorsal Spasm. By the Rev. *Richard Worthington*, M. D. 8vo. *Debrett*, London, 1792.

23. A Treatiſe on the Management of Female Complaints, and of Children in early Infancy. By *Alexander Hamilton*, M. D. Profeſſor of Midwifery in the Univerſity, and Fellow of the Royal College of Phyſicians, and of the Royal Society of Edinburgh. 8vo. *Murray*, London, 1792.

24. Eſſay on Pulmonary Conſumptions, including the Hiſtories of ſeveral remarkable Inſtances of Recovery from the moſt alarming Stages of the Diſorder, by an improved Method of Treatment. By *William May*, M. D. Member of the Royal College of Phyſicians, London; Fellow of the London Medical Society; late one of the Phyſicians to the Royal Univerſal Diſpenſary, London. 8vo. *Cadell*, London, 1792.

25. A Treatiſe on the Gout; wherein is delivered a new Idea of the proximate Cauſe, and conſequent Means of Relief; written with a View to excite farther Reſearches into the Nature, and to leſſen preſent Reſerve in the Treatment of that Diſeaſe. By *Thomas Jeans*, M. D. 8vo. *Cadell*, London, 1792.

26. An Inquiry into the Nature and Cauſes of Sickneſs in Ships of War; ſhewing the Error of its being chiefly aſcribed to Maritime

Diet, and that it cannot be prevented by the Acids ſo generally recommended; by what Means that Prevention may be moſt effectually attained, and with leaſt Expence to the State. To which are added, a Review of Sir John Pringle's Diſcourſe on preſerving the Health of Mariners, with other Medical Diſquiſitions; including Remarks on the New Diſpenſatory of the London College of Phyſicians. By *William Renwick*, Surgeon in the Royal Navy. 8vo. *Evans*, London, 1792.

27. A Commentary on Apoplectic and Paralytic Affections, and on Diſeaſes connected with the Subject. By *Thomas Kirkland*, M. D. Member of the Royal Medical Society, Edinburgh; of the Medical Society, London; and of the Agricultural Society, Leiceſterſhire. 8vo. *Dawſon*, London, 1792.

28. Obſervations on Maniacal Diſorders. By *William Pargeter*, M. D. 8vo. *Murray*, London, 1792.

29. A practical Eſſay on Diſeaſes of the Viſcera, particularly thoſe of the Stomach and Bowels, the Liver, Spleen, and urinary Bladder: In which their Nature, Treatment, and Cure, are clearly pointed out and explained. By *John Leake*, M. D. Member of the Royal College

College of Phyſicians, London. 8vo. *Evans*, London, 1792.

30. Chirurgical Obſervations relative to the Epiphora or Watery Eye, the ſcrofulous and intermittent Ophthalmy, the Extraction of the Cataract, and the Introduction of the Male Catheter. By *James Ware*, Surgeon. 8vo. *Dilly*, London, 1792.

31. The general and particular Principles of Animal Electricity and Magnetiſm, &c. in which are found Dr. Bell's Secrets and Practice, as delivered to his Pupils in Paris, London, Dublin, Briſtol, Glouceſter, Worceſter, Birmingham, Wolverhampton, Shrewſbury, Cheſter, Liverpool, Mancheſter, &c. &c. Shewing how to magnetiſe and cure different Diſeaſes; to produce Criſes, as well as Somnambuliſm or Sleep-walking; and in that State of Sleep to make a Perſon eat, drink, walk, ſing, and play upon any Inſtruments they are uſed to, &c.; to make Apparatus and other Neceſſaries to produce magnetical Facts; alſo to magnetiſe Rivers, Rooms, Trees, and other Bodies, animate and inanimate; to raiſe the Arms, Legs, of a Perſon awake, and to make him riſe from his Chair; to raiſe the Arm of a Perſon abſent from one Room to another; alſo to treat him at at a Diſtance. All the new Experiments and Phenomena are explained. By Monſieur le Docteur *Bell,* Profeſſor of that Science, and Member of the Philoſophical Harmonic Society at Paris, Fellow Correſpondent of M. le Court de Gibelin's Muſeum, and the only Perſon authorized by Patent from the firſt Noblemen in France to teach and practiſe that Science in England, Ireland, &c. 8vo. *Richardſon,* 1792.

32. The Plan adopted by the Governors of the Middleſex Hoſpital for the Relief of Perſons afflicted with Cancer: with Notes and Obſervations. By *John Howard,* Surgeon. 8vo. *Debrett,* London, 1792.

33. On the Properties of Matter, the Principles of Chemiſtry, and the Nature and Conſtruction of aeriform Fluids or Gaſes: in which the Abſurdities of the Theories hitherto advanced, and generally received, reſpecting thoſe Subſtances are fully expoſed; and ſuch an Explanation of them given as Reaſon naturally points out, and every Obſervation fully confirms. By *E. Peart,* M.D. and correſponding Member of the Medical Society of London. 8vo. *Miller,* London, 1792.

3. A Botanical Arrangement of Britifh Plants, including the Ufes of each Species in Medicine, Diet, rural Œconomy, and the Arts. With an eafy Introduction to the Study of Botany, &c. The Second Edition. By *William Withering*, M. D. F. R. S. 3 Vols. 8vo. *Robinfons*, London, 1787-92.

35. A compendious Syftem of the Theory and Practice of modern Surgery, arranged in a new nofological and fyftematic Method, different from any yet attempted in Surgery. In the Form of a Dialogue. By *Hugh Munro*, Surgeon; Prefident of the Chirurgo-Phyfical, and Extraordinary Member of the American Phyfical Societies of Edinburgh. 8vo. *Richardfon*, London, 1792.

36. Memoirs of the Medical Society of London, inftituted in the Year 1772. Vol. III. 8vo. *Dilly*, London, 1792.

37. The Chirurgical Works of *Benjamin Gooch*, Surgeon: A new Edition, with his laft Corrections and Additions. In three Volumes. 8vo. *Johnfon*, London, 1792.

38. Obfervations on the Nature and Method of Cure of the Phthifis Pulmonalis, or Confumption of the Lungs; from Materials left by the late *William White*, M. D. F. A. S. and now publifhed lished by *A. Hunter*, M.D. F.R.S. L. and R.S. E. 8vo. York, 1792.

39. Essays on the Practice of Midwifery, in natural and difficult Labours. By *William Osborn*, M.D. 8vo. *Cadell*, London, 1792.

40. Letters to Dr. *William Osborn*, Teacher and Practitioner of Midwifery in London, on certain Doctrines contained in his Essays on the Practice of Midwifery; from *Alexander Hamilton*, M.D. F.R.S. Ed. Professor of Midwifery in the University, and Fellow of the Royal College of Physicians, Edinburgh. 8vo. *Murray*, London, 1792.

41. A View of the Diseases of the Army in Great Britain, America, the West Indies, and on Board of King's Ships and Transports, from the beginning of the late War to the present Time; together with monthly and annual Returns of the Sick, and some Account of the Method in which they were treated in the twenty-ninth Regiment, and the third Battalion of the sixtieth Regiment. By *Thomas Dickson Reide*, Surgeon to the first Battalion of the first (or royal) Regiment of Foot. 8vo. *Johnson*, London, 1792.

42. A Treatise on the regular, irregular, atonic, and flying Gout; containing many new

Reflections on its Caufes, and Management under various Circumftances and Conftitutions: with the excellent Effects of the Muriatic Acid in the Relief of that Diforder. By *William Rowley*, M. D. Member of the Univerfity of Oxford, the Royal College of Phyficians in London, and Phyfician to the St. Mary-le-bone Infirmary. 8vo. *Wingrave*, London, 1792.

43. Sketches of Facts and Opinions refpecting the Venereal Difeafe. By *William Houlfton*, Member of the Corporation of Surgeons, Fellow of the Society of Antiquaries, and of the Medical Society of London, and Surgeon to the Philanthropic Reform. 8vo. *Cadell*, London, 1792.

44. The Reftorer of Health, and Phyfician of Nature, exhibiting the Caufes, Prevention, and beft Methods of curing Difeafes, as at prefent adopted by the moft celebrated Phyficians, adapted for all capacities and defcriptions of men, without technical Terms or Latin Prefcriptions. By one of the Faculty. 8vo. *Richardfon*, London, 1792.

45. An Inquiry into the Nature, Caufe, and Cure of the Gout; and of fome of the Difeafes with which it is connected. By *John Gardiner*, M. D. Fellow of the Royal College of Phyficians, cians, and of the Royal Society of Edinburgh. 8vo. *Robinsons*, London, 1792.

46. An Essay on the Swelling of the Lower Extremities, incident to Lying-In Women. By *Charles Brandon Trye*, Member of the Corporation of Surgeons, and Surgeon to the General Infirmary in Gloucester. 8vo. *Murray*, London, 1792.

47. Transactions of a Society for promoting Medical and Chirurgical Knowledge. 8vo. *Johnson*, London, 1792.

48. An Essay upon Single Vision with two Eyes; together with Experiments and Observations on several other subjects in Optics. By *William Charles Wells*, M. D. 8vo. *Cadell*, London, 1792.

49 An Inaugural Dissertation * on the Disease produced by the Bite of a Mad Dog, or other Rabid Animal: submitted to the Examination of the Rev. *John Ewing*, S. T. P. Provost, the Trustees and Medical Faculty of the University of Pennsylvania, on the eleventh day of May 1792, for the degree of Doctor of

* This Dissertation has been republished, with a Preface and Appendix, by *J. C. Lettsom*, M. D. F. R. S. 8vo. *Dilly*, London, 1793.

Medicine, by *James Mease*, A. M. of Philadelphia. 8vo. Philadelphia, 1792.

50. An Inquiry into the remote Caufe of Urinary Gravel. By *Alexander Philip Wilfon*, M. D. Soc. Med. Edin. Soc. 8vo. Edinburgh, 1792.

51. Dermato-Pathologia; or practical Obfervations, from fome New Thoughts on the Pathology and proximate Caufe of Difeafes of the true Skin, and its Emanations, the Rete mucofum, and Cuticle. With an Appendix, containing further Obfervations on the Influence of the perfpirable Fluid in the Production of Animal Heat; and Remarks on the late Theories of Scurvy, with the particular View of recommending the Oak Bark, as a new marine Antifcorbutic, and as a probable Antifeptic in fome other putrefcent Diforders. By *Seguin Henry Jackfon*, M. D. Member of the Royal Medical Society of Edinburgh, Phyfician to the Weftminfter General Difpenfary, and to the Infirmary of Saint George's, Hanover Square. 8vo. *Robfon*, London, 1792.

52. Outlines of the Doctrines in Natural Hiftory, Chemiftry, and Economics, which, under the Patronage of the State, are now delivering in the College of New York. By *Samuel*

*Samuel Latham Mitchill*, Chem. Phyf. & Agricultur. in Acad. Columb. Prof. Societat. Americ. Philofoph. Sod. Medicin. Doct. & in Republ. Nov. Eborac. lxx vir, &c. 8vo. New-York, 1792.

53. Catalogue * of Plants, exotic and indigenous, in the Botanical Garden, Jamaica, 1792. 4to. St. Jago de la Vega.

54. Hortus Eaftenfis: or, a Catalogue † of Exotic Plants in the Garden of *Hinton Eaft*, Efq. in the mountains of Liguanea, in the Ifland of Jamaica, at the time of his deceafe. To which are added, their Englifh Names, native places of Growth, by whom introduced into the Country, and as far as can be afcertained the Epoch of their Introduction. 4to. Kingfton, 1792.

55. Surgical and Phyfiological Effays ‡. By *John Abernethy*, Profeffor of Anatomy to the Corporation of Surgeons, Affiftant Surgeon to St. Bartholomew's Hofpital, and Lecturer in

* By *Thomas Dancer*, M. D.

† For the Completion of this Catalogue (which was begun by the late Dr. *Thomas Clarke*) the Public are indebted to *Arthur Broughton*, M. D.

‡ 1. On the Lumbar Abfcefs. 2. On the Compofition and Analyfis of Animal Matter.

Anatomy and Surgery. 8vo. *Evans*, London, 1793.

56. Nature and Effects of Emetics, Purgatives, Mercurials, and Low Diet, in Diforders of Bengal, and fimilar Latitudes. By *John Peter Wade*, M. D. of the Hon. Eaft India Company's Bengal Eftablifhment. 8vo. *Murray*, London, 1793.

57. A Paper on the Prevention and Treatment of the Diforders of Seamen and Soldiers in Bengal, prefented to the Honourable Court of Eaft India Directors in the year 1791. By *John Peter Wade*, M. D. 8vo. *Murray*, London, 1793.

58. Practical Obfervations on Cancerous Complaints: with an Account of fome Difeafes which have been confounded with the Cancer. Alfo Critical Remarks on fome of the Operations performed in Cancerous Cafes. By *John Pearfon*, Surgeon of the Lock Hofpital and Afylum; and of the Public Difpenfary; Lecturer on the Principles and Practice of Surgery in London. 8vo. *Johnfon*, London, 1793.

59. Statutes and Rules for the Government of the General Kent and Canterbury Hofpital, open to the Sick and Lame Poor from any any Part of the County. 8vo. Canterbury, 1793.

60. A Treatise on Gonorrhœa Virulenta and Lues Venerea. By *Benjmin Bell*, Member of the Royal Colleges of Surgeons of Ireland and Edinburgh, one of the Surgeons to the Royal Infirmary, and Fellow of the Royal Society of Edinburgh, 2 vols. 8vo. Edinburgh, 1793.

61. Dissertatio Inauguralis de Discrimine quod Scarlatinam inter et Cynanchen malignam intercedit. Auctore *Edvardo Bradly*, Anglo. 8vo. Edin. 1791.

62. Dissertatio Inauguralis de Tetano. Auctore *Josia Clerke*, Anglo. 8vo. Edin. 1791.

63. Dissertatio Inauguralis de Concoctione Alimentorum. Auctore *Joanne Jacobo Erskine*, Jamaicensi. 8vo. Edin. 1791.

64. Dissertatio Inauguralis de Arthrodynia. Auctore *Roberto Donaldson Jackson*. 8vo. Edin. 1791.

65. Dissertatio Inauguralis de Syphilide. Auctore *Thoma Johnson*, Hiberno. 8vo. Edin. 1791.

66. Dissertatio Inauguralis de Blenorrhœa a Venere impura. Auctore *Gulielmo Lister*, Anglo. 8vo. Edin. 1791.

67. Dis-

67. Diſſertatio Inauguralis de Variolis. Auctore *Gulielmo Macdougall*, ab Inſula St. Croix. 8vo. Edin. 1791.

68. Diſſertatio Inauguralis de Hydrocephalo acuto. Auctore *Gulielmo Okely*, Anglo. 8vo. Edin. 1791.

69. Univerſa Vulneribus et Ulceribus Medendi ratio. Auctore *J. Fried. Köppen*, Med. D. et Phyſico Gubernii Roſſici Charcow. 4to. Marburg. 1789.

70. Specimen Phyſicum Inaugurale ſiſtens generalia quædam de Calore, et Modo quo ſpecifice Corporibus ineſt. Auctore *Johanne Mulder*, Franequera Friſio. 4to. Franequeræ, 1790.

71. Neurologiæ generalis Tractatus. Deſcriptio Anatomica Nervorum Lumbalium, Sacralium, et Extremitatum inferiorum, cum quatuor Tabulis linearibus et quatuor adumbratis, Authore *Johanne Leonhardo Fiſcher*, Phil. et Med. Doctore, in Theatro Anatomico Lipſienſi Profectore et Soc. Oecon. Membro. Folio. Lipſiæ, 1791.

72. Scriptores Neurologici minores ſive Opera minora ad Anatomiam, Phyſiologiam, et Pathologiam Nervorum ſpectantia. Tomus I. cum Tabulis æneis, edidit, notulis nonnullis illuſ-

illuſtravit, præfatus eſt, Indicibus auxit *Chr. Fr. Ludwig*, Prof. Lipſ. 4to. Lipſiæ, 1791.

73. Diſſertatio Medica Inauguralis de Corticis Fraxini excelſioris Natura et Viribus medicis. Auctore *Bern. Nath. Gottl. Schreger*, Cizenſe. 4to. Lipſiæ, 1791.

74. *Caroli* a *Linné*, Ord. Reg. Stellæ Polaris Equitis, Archiatri Regis Suec. Medic. et Botan. in Acad. Reg. Upſ. Profeſſor. rel. Genera Plantarum eorumque Characteres Naturales ſecundum Numerum, Figuram, Situm et Proportionem omnium Fructificationis Partium. Editio octava, poſt Reichardianam ſecunda, prioribus longé auctior atque emendatior, curante D. *Jo. Chriſtiano Dan. Schreber*, Seren. Marg. Brand. Onold. et Culmb. Conſ. Aul. Med. Bot. Hiſt. Nat. et Oecon. Prof. p. o. in Acad. Erlangenſi, &c. Vol. 2. 8vo. Francofurti ad Mœnum, 1791.

75. *Jo. Jac. Pauli Moldenhawer* Tentamen in Hiſtoriam Plantarum Theophraſti. 8vo. Hamburgi, 1791.

76. Catalogus Plantarum Horti Botanici Carolſruhani ſecundum Syſtematis Vegetabilium *Caroli* a *Linné* Editionem decimam quartam. 8vo. Carolſruhæ, 1791.

77. Pharmacopœia Caſtrenſis Boruſſica. Auctore *Jo. Andrea Riemer*, M. D. Regiorum Exercitium cituum Proto-Medico. Editio altera emendata. 8vo. Berolini, 1791.

78. Symbolæ Botanicæ, five Plantarum, tam earum quas in Itinere, imprimis Orientali, collegit *Petrus Forſkål*, quam aliarum, recentius detectarum, exactiores Deſcriptiones, nec non Obſervationes circa quaſdam Plantas dudum cognitas; Auctore *Martino Vahl*, Profeſſore Regio, Societatis Agrariæ Taurinenſis, Phyſico-Botanicæ et Georgophilorum Florentinorum, &c. Sodali. Folio. Hauniæ, 1791. c. tabulis æri inciſis.

79. Icones Plantarum Syriæ rariorum, Deſcriptionibus et Obſervationibus illuſtratæ, Auctore *Jacobo Juliano la Billardiere*, M. D. Decas ſecunda. 4to. Pariſiis, 1791.

80. Tabula Plantarum Fungoſarum. Auctore *Joann. Jacob. Paulet*, Doct. Med. Paris. Acad. Med. Madritenſ. Socio, &c. 4to. Pariſiis, 1791.

81. Obſervationes Botanicæ quibus Plantæ Indiæ Occidentalis aliæque Syſtematis Vegetabilium Ed. XIV. illuſtrantur earumque Characteres paſſim emendantur, cum Tabulis æneis. Auctore *Olavo Swartz*, M. D. Muſ. Reg. Suec. Præf. Acad. Cæſar. Nat. Curioſ. Reg. Holmienſ.

nec nec non Societ. Linnæan. Lond. et Physiogr. Lond. Goth. Sodali. 8vo. Erlangæ, 1791.

82. De Febribus Tentamina duo, scripsit *Franciscus Schraud*, Hungarus Pestanus, M.D. Comitatuum Csongrad et Csanad et R. Comitis Szegediensis Medicus. 8vo. Viennæ, 1791.

83. Dissertatio Medica de Inflammatione conglutinante quam Præside Doct. *Gabr. Er. Haartman*, Med. Pract. Prof. Reg. et Ord. publico Examini offert *Ulricus Pryss*, Phil. Mag. et Med. Licent. 4to. Aboæ, 1791.

84. *Bernardi Albini*, Med. quondam Profess. in Acad. Lug. Batav. Causæ et Signa Morborum. Tom. I. Causæ et Signa Febrium continuarum et intermittentium. 8vo. Danzig, 1791.

85. Dissertatio Inauguralis medica de Medicamento nov-antiquo *Tebaschir* dicto; Auctore *Maxim. Stanisl. Joseph. Lüdgers*, Hildesiensi. 8vo. Gottingæ, 1791.

86. *Ern. Fried. Christ. Graf*, Regiomonto-Franci, Dissertatio de Sensatione et Irritatione. 4to. Jena, 1791.

87. *Johannes Ludov. Klohss*, Servesta Anhaltinus, de Paracentesi Vesicæ urinariæ per Intestinum Rectum. 8vo. Jena, 1791.

88. Dissertatio Inauguralis medica sistens quædam de Lingua ut signo; quam Præside

*Ern.*

*Ern. Ant. Nicolai* publicé defendet *Johannes Christianus Graf*, Regiomonto-Francus. 4to. Jena, 1791.

89. *Lud. Castringius*, Marca-Guestphalus, de Rationibus Sectionem Cæsaream in Usum vocandi. 8vo. Jena, 1791.

90. *Stephani Lumnitzeri*, M. D. Flora Posoniensis, exhibens Plantas circa Posonium sponte crescentes, secundum Systema sexuale Linneanum digestas. 8vo. Lipsiæ, 1791.

91. Dissertatio Inauguralis de Nitri Vi gelante. Auctore *Christiano Friderico Quandt*. 4to. Jena, 1791.

92. Dissertatio Inauguralis medica de Hæmorrhagia Narium, præsertim Respectu semiotico: Auctore *Georgio Andr Riederer*, Altorfino. 8vo. Altorf. 1791.

93. De rebus ex Homero medicis Epistola qua Viro illustri *Jo. Gottfried Leonhardi*, Serenissimi Elect. Saxon. a Consil. Aulæ et Archiatro, Nomine nonnullorum Fautorum, Amicorum, Auditorumque Diem natalem et Munera ejus nova splendidissimaque gratulatur *Davides Gottlob Wolf*, A. A. L. M. et Rev. Min. Cand. 4to. Wittenberg. 1791.

94. Dissertatio inauguralis de Hæmorrhagiis Uteri nocivis; Auctore *Henrico Ludovico Henke*, Hildesiensi. 4to. Erfurt. 1791.

95. Nevro-

95. Nevro-Encefalotomia *. 8vo. Pavia, 1791.

96. Hiſtoria Sectæ Medicorum pneumaticorum; Auctore *Io. Carolo Oſterhauſen*, M. D. 8vo. Altdorf. 1791.

97. De Exploratione Obſtetricia Diſquiſitio brevis, Auctore *Frid. Henſler*, Med. D. 8vo. Altona, 1791.

98 ΝΙΚΑΝΔΡΟΥ ΑΛΕΞΙΦΑΡΜΑΚΑ. *Nicandri* Alexipharmaca, ſeu de Venenis in Potu Cibove Homini datis eorumque Remediis Carmen, cum Scholiis Græcis & *Eutecnii* Sophiſtæ Paraphraſi Græca. Ex Libris ſcriptis emendavit, Animadverſionibuſque et Paraphraſi Latina, illuſtravit *Io. Gottl. Schneider*, Saxo, Eloqu, et Phil. Prof. in Viadrina Univerſ. 8vo. Halæ, 1792.

99. *Fr. Stephani* M. et Ph. D. Prof. Bot. et Chem. Soc. Hal. Sod. Enumeratio Stirpium Agri Moſcuenſis. 8vo. Moſcow, 1792.

100. *Caroli* a *Linné*, M. D. Equ. Aur. de Stella Pol. Archiatri Reg. Suec. Med. & Bot. Prof. Upſ. rel. Prælectiones in Ordines naturales Plantarum; e proprio et *Io. Chr. Fabricii*, Prof. Kil. Mſto. edidit *Paulus Diet. Giſeke*, M. D. in Gymnaſ. Hamb. Phyſ. Prof. et Bib-

* Auct. *Vinc. Malacarne*.

liothecarius, Acad. Imp. Nat. Cur. Inſtit. Reg Hiſt. Goett. et Soc. Elect. Oecon. Lips. Sod. Acceſſit uberior Palmarum et Scitaminum Expoſitio præter plurium novorum Generum Reductiones, cum Mappa geographico-genealogica Affinitatum Ordinum, et aliquot Fructuum Palmarum Figuræ. 8vo. Hamburgi, 1792.

101. Amphibiorum Phyſiologiæ Specimen alterum, Hiſtoriam et Species Generis Stellionum ſeu Geckonum ſiſtens, Auctore *Io. Gottl. Schneider*, Saxo, Eloquent. et Philolog. Profeſſor. 4to. Trajecti ad Viadrum, 1792.

102. Obſervationes Nevrologicæ ex Anatome comparata, Auctore *Johanne Godofredo Ebel*, Medicinæ Doctore. 8vo. Frankfurti ad Oderam, 1792, c. tab. æneis.

103. Icones Plantarum rariorum; delineavit et in Aes incidit *Henricus Schwegman*; edidit et Deſcriptiones addidit *G. Voorhelm Schneevoogt*; Scriptionem inſpexit *S. I. Van Geuns, Matth.* fil. Med. et Phil. Doct. Medicinæ, Botanices ac Phyſiologiæ Prof. Ord. in Academica Trajectina, &c, Fol. Haarlem, 1792.

104. *Bernhardi Nathanael Gottlob Schregeri*, Lib. Art. Mag. Phil. Doct. Fragmenta Anatomica et Phyſiologica. Faſciculus I. 4to. Lipſiæ, 1792. c. tab. æn.

### INDEX.

### A.

ABERNETHY, John, Surgical Essays, — 215

Abscess, Lumbar, Essay on, — ibid.

——. See *Psoas*.

Adair, William, Case in which the Brachial Artery was divided — — 21

——, Effects of Oil of Turpentine in internal Hæmorrhage, — — 25

——, Case of imperforated Anus, — 27

Albinus, Bern. de Causis et Signis Morborum — 221

Alpinus, Prosper, his Observations on the Frequency and Causes of Ophthalmia at Cairo, — 151

Amphibiorum Physiologiæ Specimen, — 224

Anatomy, Works relative to, — 218, 223, 224

Aneurism, Varicose, Case of, — — 111

Animal Magnetism, Work relative to, — 208

Anus, imperforated, Case of, — — 27

Apoplexy, Work relative to, — — 207

Army, Work on the Diseases of, — — 211

Arsenic, given in India as a Remedy for Elephantiasis 172

——, recommended as an external Application in some cutaneous Disorders, — — 174

Artery, Brachial, Case in which it was wounded, 21

Arthrodynia, Dissertation on, — — 217

Ash Tree, Bark of, Treatise on, — — 219

At'har Ali Khan, on the Cure of Elephantiasis, 168

### B.

Beddoes, Dr. Thomas, Case of Phlegmonic Inflammation, 148

Bell, Benjamin, on Gonorrhea and Lues, — 217

——, Dr. on Animal Magnetism — — 208

Bengal, Works relative to the Diseases of, — 216

Bergman, Sir T. Physical and Chemical Essays, 203

Bezoar Stones, described, — — 33

——, in what Animals produced, — 34

Billardiere, J. J. la, Icones Plantarum Syriæ rariorum 220

Bladder, Dissertation on the Puncture of through the Rectum, 221

Blagden, R. B. Case of Gutta Serena, — 126
Boag, W. on the Fevers and Dysentery of hot Climates, 1
Botany, Introduction to, — — 204
Brachial Artery, Case in which it was wounded, 21
Bradly, Edv. de Scarlatina et Cynanche Maligna, 217
Broughton, Dr. A. Hortus Eastensis, — 215

C.

Cæsarean Section, Dissertation on, — 222

Calculus in general, Obs. on its Formation, — 41

——, urinary, said to occur rarely in warm Climates, 47

——, intestinal, Symptoms of, and Mode of treating, 54

————, Chemical Analysis of, — 75

Cancer, Works relative to, — 201, 209, 216

Carlsruhe, Catal. of the Botanic Garden at, — 219

Castringius, Lud. de Sectione Cæsarea, — 222

Cataract, Extraction of, Work relative to, — 208

Catheter, Male, Introduction of, Work relative to, *ibid.*

Caustics, good Effects of, in White Swellings of the Joints, 157

Chemistry, Annals of, — — 203

Children, Works relative to the Management of, 200, 206

Clarke, Dr. T. Hortus Eastensis, — 215

Clarkson, Mr. a Fact from his Essay on the Impolicy of the Slave Trade, quoted, — — 152

Clerke, Josias, de Tetano, — — 217

Climates, hot, Obs. and Works relative to the Diseases of, 1, 216

Clothing, modern, Essay on, — — 204

Concoction of Food, Dissert. on, — 217

Consumption, pulmonary, Essays on, — 206, 210

Copland, Peter, Cases of Menorrhagia, — 118

Corp, Dr. on the Effects of the Mind on the Body, 201

Crowther, B. on the Use of Caustics in White Swellings, 157

D.

Dancer, Dr. Tho. Catalogue of Plants, — 215

Davidson, William, on pulmonary Hæmorrhage, 129

Dictionary, Medical, — — 202

Dorsal Spasm, Treatise on, — — 205

Dysentery of hot Climates, Observations on, — 1

————, Use of Mercury in, 15

———— Opium in, — 17

E. East

E.

Eaft, Hinton, Catalogue of his Exotic Plants, 215
Ebel, J. G. Obferv. Nevrologicæ, —— 224
Electricity, Work relative to, — — 202
Elephantiafis, Obfervations on, —— 168
————, how named by the Arabs and Indians, 169
————, not to be confounded with the fwelled Legs defcribed by the Arabian Phyficians, — *ibid.*
Epiphora, Work relative to it, —— 208
Erfkine, Jo. Jac. de Concoct. Aliment. — 217
Extra-uterine Geftation, Cafe of, — — 203

F.

Ferriar, John, Medical Hiftories, —— 205
Fevers of hot Climates, Obf. and Works relative to, 1, 216
Fifcher, Joh. Leon. de Neurologia, —— 218
Flora Pofonienfis, — —— 222
Foot, Jeffe, on the Lues Venerea, —— 204
Forfkal, Petr. Defcript. of the Plants collected by him, 220
Fraxinus Excelfior. See *Afh Tree.*

G.

Gaitfkell, W. Obfervations on Calculi, — 31
Gardiner, Dr. John, on the Gout, —— 212
Garnett, Dr. T. on the Mineral Waters of Harrowgate, 205
Gifeke, P. D. See *Linné.*
Gonorrhœa, Treatife on, — —— 217
Gooch, Benj. Chirurgical Works of, — 210
Gout, Treatifes on, — — 206, 211, 212
Graf, Ern. F. Chr. de Senfatione et Irritatione, 221
Gravel, urinary, Work relative to, —— 214
Griffith, R. on a Reproduction of the Sphincter Ani, 204
Grigg, John, Advice to the Female Sex, — 200
Gutta Serena cured by a Mercurial Snuff, — 126

H.

Hæmorrhage, internal, Effects of Oil of Turpentine in, 25
———— of the Nofe, Semeiological Differtation on, 222
————, Uterine. See *Menorrhagia.*
————, pulmonary, Remarks on, — 129
Hamilton, Dr. Alex. on Female Complaints, 206
———— Letters to Dr. W. Ofborn, 211

Hamilton, Dr. R. on Scrophula, —— 201

Harrowgate, Treatiſe on the Waters of, — 205

Haſſelquiſt, his Hypotheſis of the Cauſe of the frequency of Ophthalmia, in Egypt, controverted, — 150

Health, the Reſtorer of, a Work ſo entitled, — 212

Heat, Diſſertation on, ———— 218

Heat and Cold, Remarks on certain Effects of on the living Syſtem, ——— ——— 148

Henke, H. L. de Hæmorrhagiis Uteri, — 222

Henſler, F. de Exploratione Obſtetricia, — 223

Homer, Work on the Medical Obſ. in the Writings of, 222

Houlſton, W. on the Venereal Diſeaſe, — 212

Howard, John, Plan for the Relief of Cancers, 209

Hunter, Dr. A. his Edition of Dr. White's Obſ. on Phthiſis, 210

Hydrocephalus, Diſſertation on, ——— 218

Hydrophobia, Work relative to, ——— 213

I.

Jackſon, Dr. S. H. on Diſeaſes of the Skin, 214

———, Robertus Donaldſon, de Arthrodynia, 217

Jamaica, Catalogues of Plants in, ——— 215

Jatamanſi, a Species of Valerian growing in India, ſuppoſed to be the Spikenard of the Ancients, — 184

Jeans, Dr. Tho. on the Gout, ———— 206

Inflammation, adheſive, Diſſertation on, ——— 221

—————, phlegmonic, Caſe of, ——— 148

————— of the Eyes, in hot Climates, a frequent Complaint, ——— ——— 149

——————————————, how accounted for by Haſſelquiſt and Alpinus, — 150, 151

——————————————, ſuppoſed to originate from the great Irregularity between the Temperature of the Night and the Day, — — 152

Johnſon, Thomas, de Syphilide, ——— 217

Jones, Sir William, on Elephantiaſis, — 168

——————, Obſ. on the Weights of the Hindus, 172

——————, on the Spikenard of the Ancients, 180

Irritation, Diſſertation on, ——— 221

K.

Kent and Canterbury Hoſpital, Rules of, — 216

Kirkland, Dr. Tho. on Apoplexies and Palſies, 207

Klohſs,

Klohss, J. L. de Paracentesi Vesicæ per Intest. Rectum, 222

Koppen, J. F. de Vulneribus et Ulceribus, — 218

L.

Leake, Dr. John, on Diseases of the Viscera, — 207

Lettsom, Dr. J. C. his Edition of a Work on the Rabies Canina — — 213

Linné, Carol. a, Genera Plantarum curâ Schreberi, 219

——————, Prælect. in Ord. nat. Plant. curâ Gisekii, 223

Lister, Gul. de Blenorrhœa, — 217

Lüdgers, M. S. J. de Medicamento *Tebaschir* dicto, 221

Ludwig, C. F. Scriptorum Neurolog. Thesaur. 218

Lues Venerea, Works on, — 204, 212, 217

Lumnitzerus, Steph. Flora Posoniensis, — 222

M.

Macdougall, Gul. de Variolis, — 218

Macie, J. L. Experiments on Tabasheer, — 193

Madness, Works relative to, — 201, 207

Malacarne, Vinc. Nevro-Encefalotomia, — 223

Mania. See *Madness*.

Mariners, Work on the Diseases of, — 206

Mather, Alex. on the Effects of Opium in Retention of Urine, — — 102

——————, Case of monstrous Birth, — 107

Matter, Animal, Essay on the Composition and Analysis of, 215

May, Dr. W. on Pulmonary Consumptions, — 206

Mease, James, on the Rabies Canina, — 213

Medical Society, in London, Memoirs of, — 210

——— and Chirurgical Society, in London, Transactions of, — — 213

Menorrhagia, Cases of, cured by opiate Clysters, 118

——————, Dissertation on, — 222

Mercury, Observations on its Use in Fevers and Dysenteries in hot Climates, — — 1

——————————— the mode of administering it, 15

Mercurial Snuff, Efficacy of, in Gutta Serena 126

Midwifery, Works relative to, 200, 206, 211, 213, 223

Mitchill, Dr. S. L. Outlines of Nat. Hist. and Chemistry, 214

Moldenhawer, J. J. P. Tentam. in Hist. Plant. Theophrasti, 219

Monstrous Birth, Case of, — 107

Moscow, Enumeration of the Plants of, — 223

Motherby, Dr. G. Medical Dictionary, — 202

Munro, Hugh, Compendium of Surgery, — 210

Muscles, Human, History of, — 202

N.

Nardus Indica. See *Spikenard.*

Nevro-Encefalotomia, — 223

Neurology, Treatise on, — 218, 224

Nicandri Alexipharmaca, — 223

Nitre, Dissertation on the freezing power of, — 222

O.

Oak Bark, recommended as a Marine Antiscorbutic, 214

Okely, Gul. de Hydrocephalo, — 218

Ophthalmy, scrofulous, Work relative to, 208

Opium, Efficacy of, in Retention of Urine, 102

———— Menorrhagia, — 118

Osborn, Dr. W. on the Practice of Midwifery, — 211

————, Letters to, — *ibid.*

Osterhausen, I. C. Hist. Sectæ Med. Pneumaticorum, 223

P.

Palsies, Work relative to, — 207

Pargeter, Dr. W. on Maniacal Disorders, — 207

Park, H. Case of Varicose Aneurism, — 111

Paulet, J. J. Tabula Plantarum Fungosarum, — 220

Pearson, John, on Cancerous Complaints — 216

Peart, Dr. E. on Electricity, — 202

———— the Properties of Matter, — 209

Perfect, Dr. W. Case of Madness, — 202

*Persian Fire*, a Name given to the Lues Venerea in Hindostan, — — 175

Pharmacopœia, of London, Analysis of, — 205

————, Castrensis Borussica, — 219

Plants, British, Botanical Arrangement of, — 210

————, of the West Indies, Observations on, 220

————, Fungous. See *Paulet.*

————, rare, Figures of, — 224

————, of Syria. See *Billardiere.* —

Pneumatic Sect of Physicians, History of, — 223

Prescriptions,

Prefcriptions, Medical, Collections of, 202, 204
Pryfs, Ulricus, de Inflammatione conglutinante, 221
Pfoas Abfcefs, Cafe of, ——— 138

Q.

Quandt, Chr. Frid. de Nitri Vi gelante, —— 222

R.

Rachitis, Work relative to it, —— 201
Reide, T. D. on the Difeafes of the Army, — 211
Renwick, W. on Sicknefs in Ships of War, 206
Riederer, Geo. And. de Hæmorrhagia Narium, 222
Riemer, J. And. Pharmac. Caftrenfis Boruffica, 219
Rowley, Dr. W. on the Gout ——— 212

S.

Saunders, Samuel, Introduction to Botany, — 204
Scarlatina & Cynanche Maligna, Differt. on the diftinction between, —— — — 217
Scirrhus, Work relative to it, —— —— 201
Schneevoogt, G. V. defcript. plant. rariorum, 224
Schneider, J. G. his edition of the Alexipharmaca Nicandri, 223
——————, Amphib. Phyfiolog. Specimen, 224
Schraud, Franc. de Febribus, —— 221
Schreber, J. C. D. his edition of the Genera Plantarum Linnæi, —— ——— —— 219
Schreger, B. N. G. de Cortice Fraxini excelfioris, *ibid.*
Scrophula, Work relative to it, — — 201
Senfation, differtation on, —— —— 221
Sinapifms, recommended in dropfy of the joints, 161
Skin, Work on the difeafes of, —— 214
Smith, Dr. Hugh, Formulæ Medicamentorum, 204
Spafm dorfal, Treatife on, — — 206
Small Pox, differtation on, —— 218
Sphincter ani, Work relative to the reproduction of, 204
Spikenard of the ancients, obs. on. — 180
——————————, mentioned by Diofcorides, *ibid.*
——————————, not known with certainty to Linnæus, —— — *ibid.*
——————————, not, as hath been fuppofed, a fpecies of Andropogon — 189, 190
——————————, but a fpecies of Valerian, 190
——————————, Botanical defcription of, 191
Snuff, Mercurial, Efficacy of, in a cafe of Gutta Serena, 126

Stephanus, Fr. Flora Moscuensis, — — 225

Surgery, compendious System of, — — 210

Swartz, Olavus, de Plantis Indiæ occidentalis, 220

Syphilis. See *Lues Venerea*,

T.

Tabasheer, Chemical Experiments on, — 193

———, specific Gravity of, — 195

———, found to be identical with siliceous earth, 196

———, in a green Bamboo growing in England, 199

———, Dissertation on, — — 221

Tetanus, Dissertation on, — — 217

Thomas, R. on the Diseases of warm Climates, 201

Tongue, as a sign in Diseases, Dissertation on, 221

Trye, C. B. on the swelling of the lower Extremities in Lying-in Women, — — 213

Tunbridge Wells, Analysis of the Waters of 204

Turnbull, William, Case of Extra-Uterine Gestation 203

Turpentine, Oil of, its effects in internal Hæmorrhage, 25

U.

Vahl, Martin. Symbolæ Botanicæ, — 220

Varicose aneurism, Case of, — 111

Vaughan, Dr. Walter, Essay on modern Clothing, 204

Viscera, Work on the Diseases of, — 207

Vision, single, with two Eyes, Essay on, — 213

Urine, Case of Retention of, cured by Opium, 102

W.

Wade, Dr. J. P. on Emetics, &c. in Diseases of Bengal, 216

———, the Diseases of Seamen and Soldiers in Bengal, — — *ibid.*

Wallis, Dr. G. Medical Dictionary, — 202

Ware, James, on the Epiphora, — 208

Wells, Dr. W. C. on single Vision with two Eyes, 213

White, Dr. R. Analysis of the London Pharmacopœia, 205

———, Dr. W. on Phthisis Pulmonalis, — 210

——— Swellings of the Joints, good Effects of Causticsin, 157

Whytt, Dr. recommends Opium in Uterine Hæmorrhage, 120

Wilson, Dr. A. P. on Urinary Gravel, — 214

Withering, Dr. William, Arrangement of British Plants, 210

Wolf, Dav. G. de Rebus ex Homero medicis, 222

Worthington, Rev. Dr. R. on the Dorsal Spasm, 205

Wounds and Ulcers, Work relative to, — 218

Wright, Thomas, History of the Human Muscles, 203

END OF THE FOURTH VOLUME.

